# Modification of polyurethane foams with zinc sulfide nanoparticles and their novel composites with multani mitti and charcoal for oil spill cleanup†

**DOI:** 10.1039/d4ra03924f

**Published:** 2024-08-13

**Authors:** Shumaila Saleem, Sadia Khalid, Aalia Nazir, Yaqoob Khan, Majid Ali

**Affiliations:** a Institute of Physics, The Islamia University of Bahawalpur Bahawalpur Pakistan; b Nanosciences & Technology Department, National Centre for Physics, Quaid-e-Azam University Campus Shahdra Valley Road Islamabad 45320 Pakistan sadia.khalid@ncp.edu.pk sadiabzu@gmail.com; c Thermal Energy Engineering Department, National University of Science and Technology (NUST) Islamabad Pakistan

## Abstract

With the rapid growth of the automobile industry, the excessive number of industrial pollutants, particularly oil spills, has become a huge threat to the natural environment. Therefore, an environmentally benign and sustainable solution is required for an effective oil spill cleanup. To enhance the sorption capacity of pristine polyurethane (PU) foam used in oil spill cleanup, ZnS nanoparticles were deposited on PU foam *via* a coprecipitation approach. Additionally, the effect of Fuller's earth, locally known as Multani Mitti (MM), and charcoal (CC) on the sorption properties of the PU foam were investigated and compared. Polyvinyl alcohol (PVA) was used as a binder during the modification procedure. The morphology, chemical composition, and thermal stability of ZnS/MM/PVA- and ZnS/CC/PVA-modified PU sorbents were characterized using X-ray diffraction (XRD), scanning electron microscopy (SEM), Fourier transform infrared (FTIR) spectroscopy, thermogravimetric analysis (TGA), and X-ray photon spectroscopy (XPS). The modified PU foam exhibited outstanding properties including a high sorption capacity, high selectivity to different types of used oils such as vegetable oil, hydraulic oil, lube oil, and gear oil, and superior reusability in comparison to pristine PU foam. ZnS/CC/PVA has a sorption capacity of 16.78 g g^−1^ while ZnS/MM/PVA exhibited a sorption capacity of 16 g g^−1^. In addition, after 10 cycles of oil sorption-squeezing experiments, the oil sorption capacity remained unchanged, and the absorbed used oil could be removed and collected by an easy squeezing procedure prior to reuse. This work reveals that the ZnS/CC/PVA- and ZnS/MM/PVA-modified PU foams have a promising potential for oil spill removal and environmental protection.

## Abstract 2

Modification of PU foam with ZnS based nanocomposites was achieved *via* simple, scalable and economical coprecipitation route. These modified PU foam exhibited high sorption capacity and mechanical stability which have promising potential for oil spill removal from the environment.

## Graphical abstract

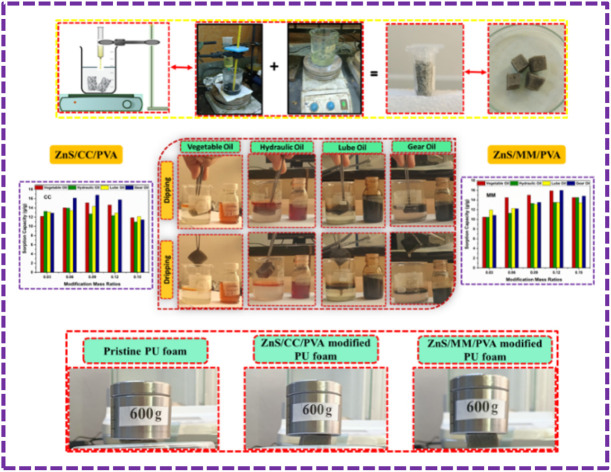

## Introduction

Accidental release of liquid petroleum hydrocarbons into the environment, usually through water, is known as an oil spill. Oil spills, becoming an everyday incident, attract significant media and official attention when they are of significant scale or occur in environmentally vulnerable areas. The petroleum from oil fields to consumers necessitates delivery through various means of transportation, including tankers, pipelines, railcars, and tank trucks. Petroleum is kept at many locations, including transfer sites, terminals, and refineries situated along its transportation path. Accidents have the potential to occur at several stages of manufacture, transit, or storage.^1,2^

Oil spills are an environmental threat that can be attributed to the extensive utilization of oil and petroleum derivatives. Globally, an estimated 20 million tons are used on a daily basis.^3^

Porous polymeric composites are regarded as highly efficient adsorption materials due to their ease of preparation and recyclability, such as polypropylene (PP)/polyester (PET) fibre,^4^ polyacrylonitrile (PAN)/reduced graphene oxide (rGO),^5^ and waste newspaper/waste polystyrene (PS).^6^ They exhibit acceptable adsorption capacity and demonstrate favourable oil selectivity, which may be achieved by appropriate adjustments to the hydrophobic and hydrophilic nature of their surface.^7–10^ Various polymer grades have been utilized as oil adsorbents, including foams,^11,12^ resins,^13,14^ sponges,^15^ and aerogels.^16,17^

Polyurethane foams have garnered significant interest in recent times for their application in oil/water separation processes^18,19^ within the field of various polymer matrices. Nevertheless, the inclusion of polar groups, such as carboxyl and amino groups, inside the PU frameworks makes these materials hydrophilic, hence affecting their selectivity and overall performance. PU foams, which include characteristics such as ultralight, open-cell structure, high porosity, and low density have been well recognized for their effectiveness as oil sorbents.^20^ Extensive studies have been conducted on the impact of the cell structure^21^ and foam density on the oil sorption capabilities of PU.^22^ Insufficient emphasis has been placed on the surface modification of PU foams with the objective of enhancing their oleophilic/hydrophobic properties for the purpose of oil spill remediation.^18^ A PU foam-based adsorbent for oil spill cleanup has been reported by Wu *et al.*, which was prepared through a novel method by treating PU foam with SiO_2_. The volume of the PU foam was 13.5 cm^3^. The sorption capacities of motor oil and diesel oil were 103 and 95 g g^−1^, respectively.^19^

Zinc sulfide (ZnS) is an n-type semiconductor that has a broad band gap (2.6–4.6 eV), strong electrical mobility, good thermal stability, non-toxicity, water insolubility, large surface area, and is relatively inexpensive. ZnS has been identified as a suitable nanomaterial for the removal of organic contaminants from wastewater.^23^

Fuller's earth (MM) is commonly used in subcontinent South Asia for cleansing skin and has been employed in various applications, owing to its notable adsorption capabilities and cost-effectiveness.^24^ Bleaching earth enhances the oil's quality and lightens the tone of any coloured oil by modifying the basic colour units in the oil without changing the chemical qualities of the oil. Additionally, it eliminates other impurities such as soap, aromatic compounds, residual metals, oxidized substances, and phospholipids.^24,25^ Charcoal/activated carbon (CC) covers a diverse array of amorphous carbon-based substances that are characterized by their high porosity and extensive inter-particulate surface areas.^26^ By increasing the adsorbent quantity and contact time, oil removal is improved by CC. With a removal efficiency of 99.67%, CC was determined to be the most effective adsorbent compared to rice hull.^27^ CC can be modified to acquire magnetic properties to facilitate cleanup collection.^28,29^

Polyvinyl alcohol (PVA) is a linear or semi-crystalline synthetic polymer that is white or creamy in colour, tasteless and odourless, nontoxic, biocompatible, and thermostable.^30,31^ It has been used as a binder in this work to improve the adherence of deposited films.

This work presents a simple, effective, and innovative approach that involves the utilization of PU foam derived from discarded laboratory materials as a sorbent for oil spills. To the best of our knowledge, ZnS/CC/PVA and ZnS/MM/PVA-modified PU foams were employed for the first time to improve the oil sorption capacity. The presence of ZnS effectively improved the oil adsorption capacity. The modified PU foam was characterized by FT-IR, SEM, TGA and XPS. Several used oils were adopted as model pollutants, and the sorption mechanism was identified. The modified PU foams can continuously, quickly and effectively separate the used oil from water. It also has an important feature for large-scale applications in oil spill cleanup.

## Experiment

### Material and methods

#### Materials

100% polyurethane foam (2 cm × 2 cm × 1 cm) was used as the sorbent. Zinc nitrate hexahydrate (Zn(NO_3_)_2_·6H_2_O), sodium sulfide (Na_2_S), Triton X-100 (TX-100) and polyvinyl alcohol (PVA) were supplied from Sigma-Aldrich. Fuller's earth (MM) and activated charcoal/carbon (CC) were locally purchased.

#### Synthesis of the ZnS/CC/PVA-modified PU foam

The PU foam was washed by an immersion process, which includes acetone and deionized (DI) water solution in an ultrasonic bath for 15 minutes. Subsequently, the foam was dried in an oven at a temperature of 60 °C for 2 hours.

**Fig. 1:**
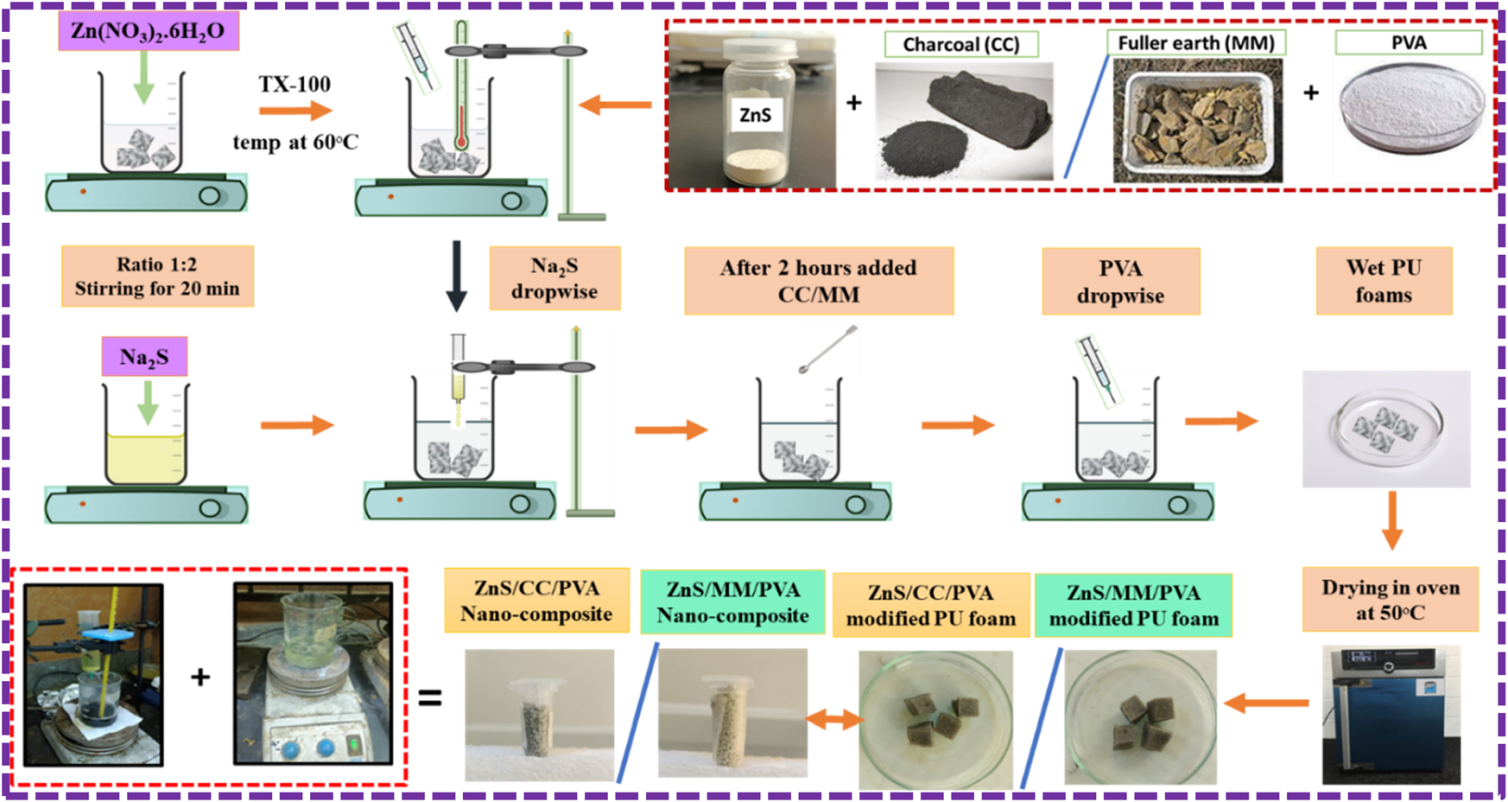
Schematic representation of the ZnS/CC/PVA and ZnS/MM/PVA nanocomposites-modified PU foams.

Modification of PU foams by ZnS/CC/PVA was carried out *via* coprecipitation method. The PU foam was coated with ZnS nanoparticles using 1 M Zn(NO_3_)_2_·6H_2_O and 2 M Na_2_S in the presence of TX-100 at 60 °C. After 2 h, the calculated amount of CC powder and PVA were added to the solution with continuous stirring for 3 h. Finally, ZnS/CC/PVA-modified PU foams and the resulting precipitates were collected and dried at 50 °C for 24 h in an oven, as shown in Fig. 1. The CC powder was used by wt%, *i.e.*, 0.03 wt%, 0.06 wt%, 0.09 wt%, 0.12 wt%, and 0.15 wt% of ZnS, and the series of modified PU foams were prepared with the sample names of CC3, CC6. CC9, CC12 and CC15, respectively.

#### Synthesis of ZnS/MM/PVA modified PU foam

The PU foam was washed by an immersion process, which included acetone and DI water solution in an ultrasonic bath for a duration of 15 minutes. Subsequently, the foam was dried in an oven at a temperature of 60 °C for 2 hours.

Modification of PU foams by ZnS/MM/PVA was carried out using the same procedure as mentioned above for ZnS/CC/PVA, and the series of modified PU foams were prepared with the sample names of MM3, MM6. MM9, MM12 and MM15.

**Fig. 2:**
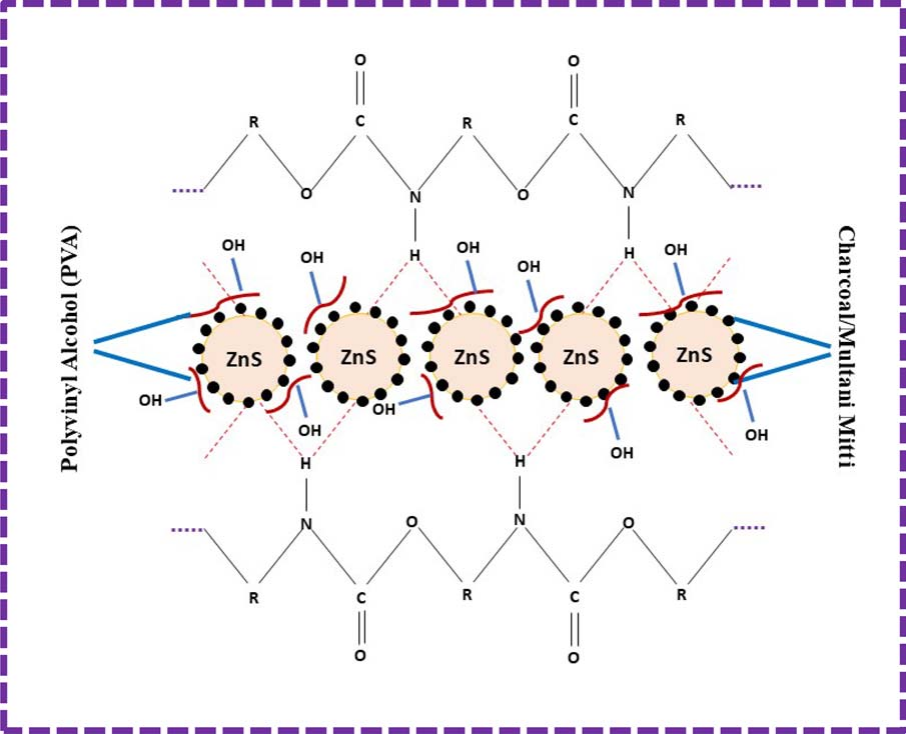
Mechanism of the ZnS/CC/PVA and ZnS/MM/PVA-modified PU foams.

The ZnS nanoparticles interacted with the PU foam. During deposition, hydrogen bonds were formed between ZnS and PU foam. The remaining void spaces were filled with the CC and MM, and PVA was added for the binding of ZnS, CC, and MM. The overall mechanism of the ZnS/CC/PVA and ZnS/MM/PVA-modified PU foams is shown in Fig. 2.

## Characterizations

### X-ray diffraction (XRD)

The XRD patterns of CC and MM were obtained by a Bruker D8 Advance X-ray diffractometer with a Cu Kα radiation wavelength (*λ*) of 1.5418 Å within the 2*θ* range of 5–70°. The Bragg equation was utilized to determine the spacing (*d*) between the diffracting planes of CC and MM:

1 2*d* sin *θ* = *nλ*

where *d* is the interplanar distance, *θ* denotes the scattering angle, *n* is a positive integer, and *λ* is the wavelength of X-rays.

The crystallite size was calculated using the Scherrer equation.2
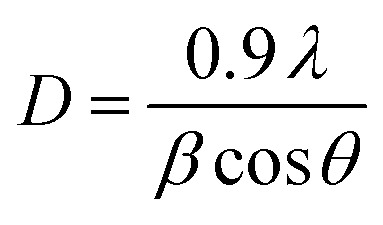
where *β* denotes the full-width at half-maximum (FWHM), and *λ* is the wavelength of the X-rays.^32^

### Field emission scanning electron microscopy (FE-SEM)

The modified PU foam structure was observed by ZEISS FE-SEM at 15 kV with an Oxford energy-dispersive X-ray spectrometer (EDS) for elemental analysis.

### Fourier transform infrared spectroscopy (FT-IR)

Fourier Transform Infrared Spectroscopy (FT-IR) measurements were performed using attenuated total reflectance (ATR) by a Bruker TENSOR 27. The spectra were measured at 4000–600 cm^−1^ and the wavenumber revolution was 4 cm^−1^.

### Thermogravimetric analysis (TGA) and differential scanning calorimetry (DSC)

TGA of ZnS/MM/PVA and ZnS/CC/PVA were performed in a nitrogen environment by METLLER Toledo TGA with a sample weight of 3.827 mg, and within the temperature range of 49–900 °C.

### X-ray photoelectron spectroscopy (XPS)

The XPS data were acquired using a Scienta-Omicron system that was fitted with a micro-focused monochromatic Al K-alpha X-ray source. The data collection was done using Matrix software, while the subsequent data analysis was carried out with CasaXPS software, using XPS fit techniques. The process of fitting the detailed spectra involved the utilization of Gaussian–Lorentzian line shape after background adjustments.

### Density

The densities of the pristine PU foam, ZnS/CC/PVA and ZnS/MM/PVA-modified PU foams were obtained by calculating the weight and volume of each respective foam sample. The samples were weighed using a high-precision analytical balance (with a readability of 0.0001 g), and their size was measured using a scale. Each group utilized different samples to determine the density (*ρ*). The density was computed using the following equation:^33^3
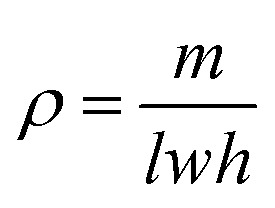
where *m*, l, *w*, and *h* are the weight, length, width, and height of the samples, respectively.

### Measurement of the oil sorption performance

The oil and other solvent absorption capacities of the adsorbent were determined. Before immersion in the liquids, the adsorbent sample mass was measured and observed. After saturation, the foam was removed and allowed to drain freely until dripping stopped. It was then placed in a pre-weighed container, and weighed and recorded. The sorption capacity (*S*_c_) was determined using the following equation:4
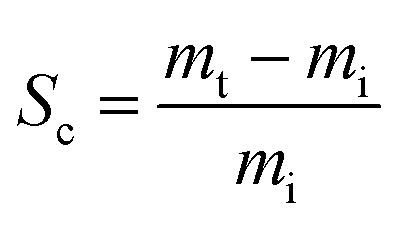
where *m*_i_ and *m*_t_ are the weights of the PU foam before and after the first absorption, respectively.

### Reusability

The reusability of the adsorbent was determined by measuring the change in the weight of the adsorbent after compressing the absorbed oils and the quantity of recovered oils.

This process was performed 10 times. The oil recovery rate (*R*%) is calculated as follows:5
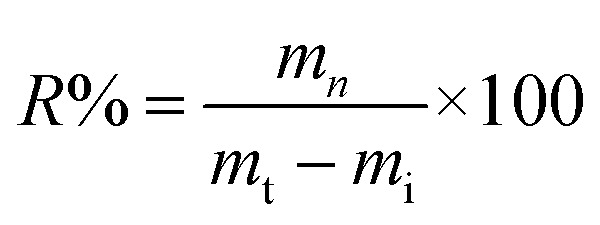
where *m*_*n*_ is the weight of the recovered oil at the nth time. All tests were performed at room temperature.^34^

## Results and discussions

### XRD

**Fig. 3:**
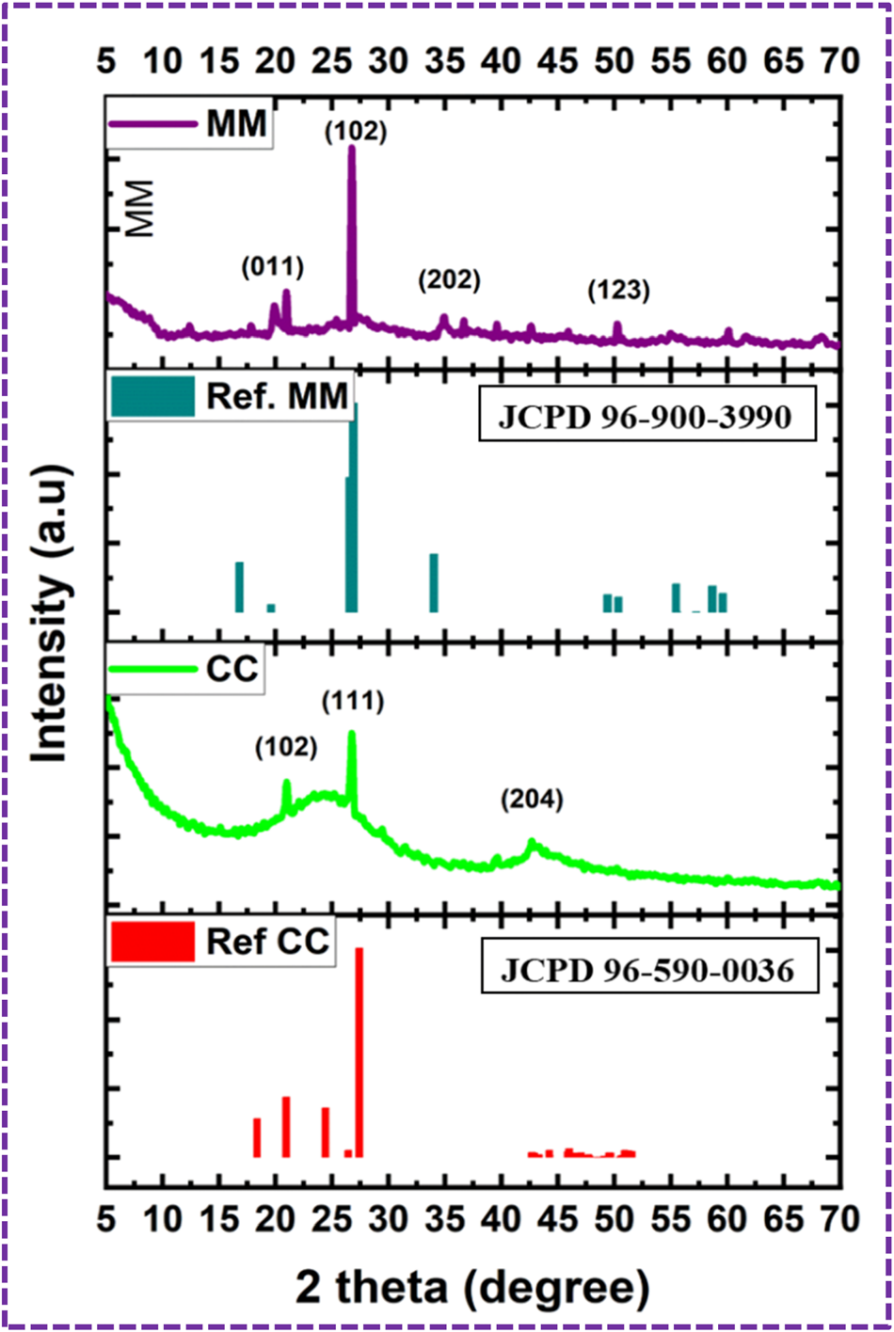
XRD patterns of CC and MM.

XRD analysis was used to investigate the crystallographic information of the CC and MM, as shown in Fig. 3. XRD analysis showed the monoclinic crystal system of CC and orthorhombic system for MM.^35^ The diffraction peaks at 20.99°, 26.79°, and 42.66°, which correspond to the (102), (111), and (204) crystal planes, respectively, are similar to the diffraction peaks of the monoclinic crystal system of CC (JCPDS 96-590-0036). In addition, the peaks at 19.9034°, 26.7382°, 34.8477°, and 50.2705° correspond to the (011), (102), (202), and (123) crystal planes of MM, respectively (JCPDS 96-900-3990). The average crystallite sizes of the CC and MM nanomaterials were calculated by Scherrer eqn (2) as 33.2 nm and 32.6 nm, respectively.

### FE-SEM

**Fig. 4:**
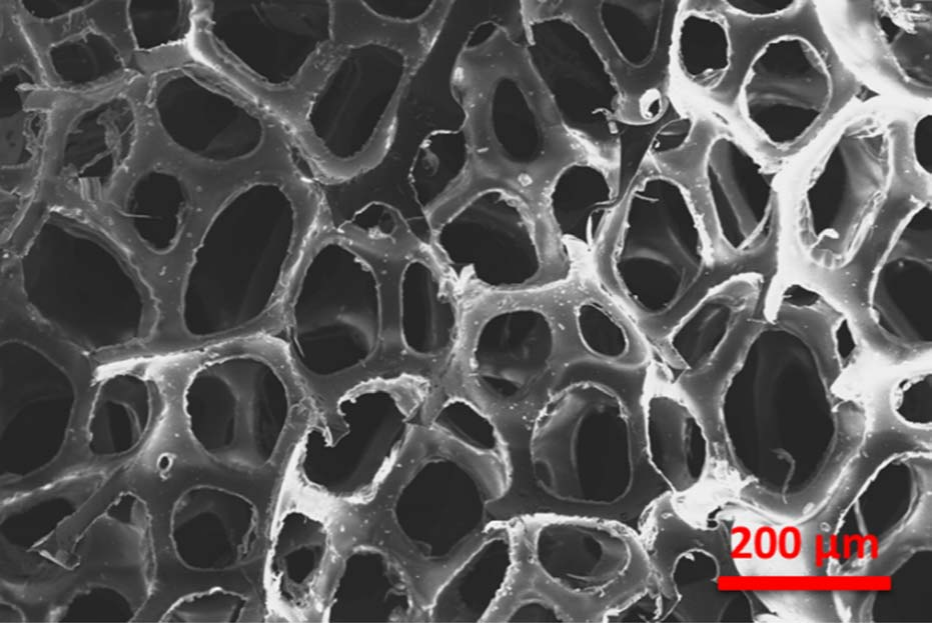
FE-SEM image of the pristine PU foam.

**Fig. 5:**
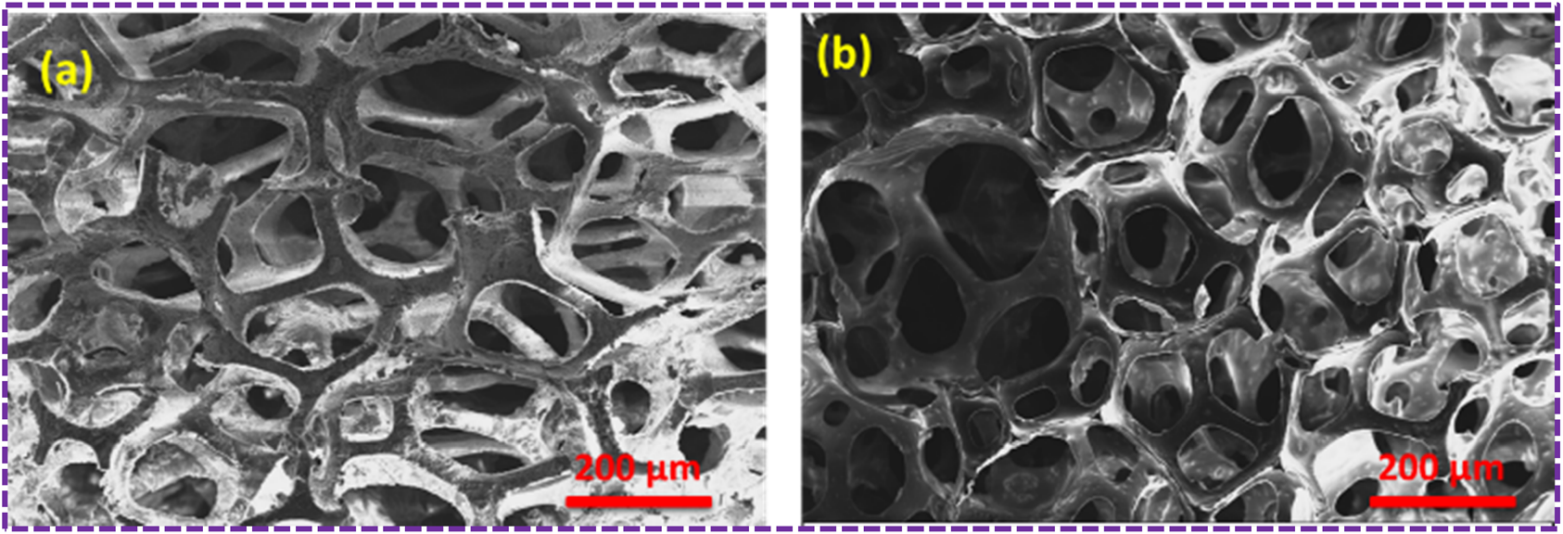
(a) SEM image of the ZnS/CC/PVA-modified PU foam before the oil sorption test. (b) SEM image of the ZnS/CC/PVA-modified PU foam after oil sorption.

The SEM images of the pristine PU foam are shown in Fig. 4. The surface modification of the PU foam with the ZnS/CC/PVA nanocomposite was analysed with FESEM/EDX before the sorption test. These foams possess void spaces and surface areas to absorb the used oil. Fig. 5a and b compares the morphology of the nanocomposite before and after the oil sorption test. After surface modification, the surface becomes rough due to the coating of a layer of ZnS/CC/PVA. Due to amplification or enlargement of the solid–liquid interactions, this introduced irregularity to the foam influences its interaction with a wetting or nonwetting liquid. The microstructure and pore shape of the modified PU foam indicate that the incorporation of ZnS/CC/PVA into the foam did not break the porous structure of the PU foam.^36^Fig. 5b shows the modified PU foam before and after 10 cycles of the oil sorption test, and the surface stability of the PU foam is quite evident.

**Fig. 6:**
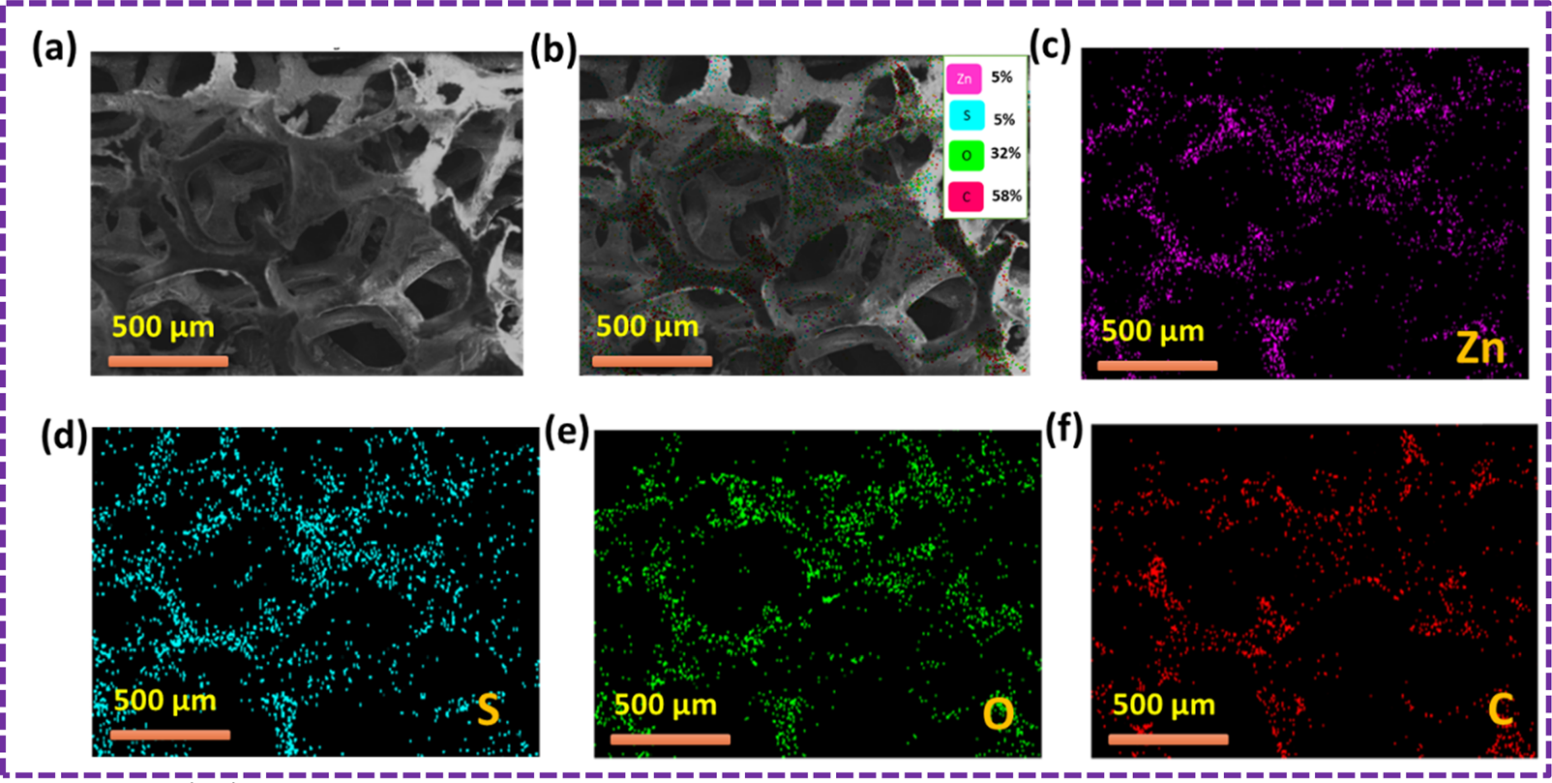
SEM image of the ZnS/CC/PVA-modified PU foam; (a and b) EDX mapping and elemental analysis, including elemental analysis of (c) Zn, (d) S, (e) O, and (f) C before the oil sorption test.

**Fig. 7:**
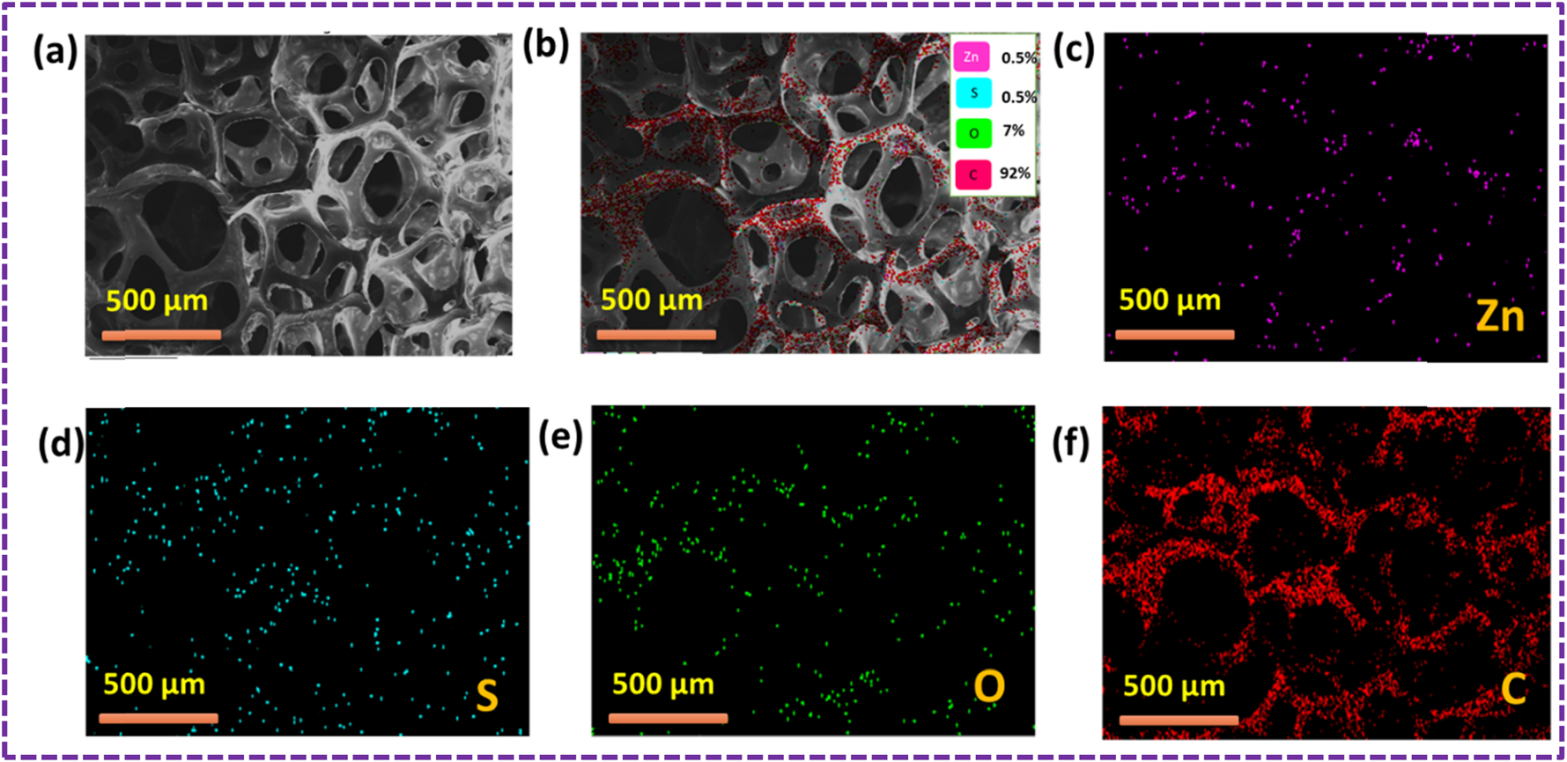
SEM image of the ZnS/CC/PVA-modified PU foam; (a and b) EDX mapping and elemental analysis, including elemental analysis of (c) Zn, (d) S, (e) O, and (f) C after the oil sorption test.

The outcomes of the elemental mapping in Fig. 6 reveal the presence of Zn, S, C, and O, indicating the existence of the ZnS/CC/PVA nanoparticles prior to the oil sorption test. The nanocomposite exhibited a uniform distribution of Zn, S, C, and O. The results of the oil sorption test, as depicted in Fig. 7, reveal the presence of Zn, S, C, and O from elemental mapping. This suggests the existence of ZnS/CC/PVA nanoparticles. The nanocomposite exhibited a uniform dispersion of Zn, S, C, and O atoms. The carbon content is elevated after conducting an oil sorption test.

**Fig. 8:**
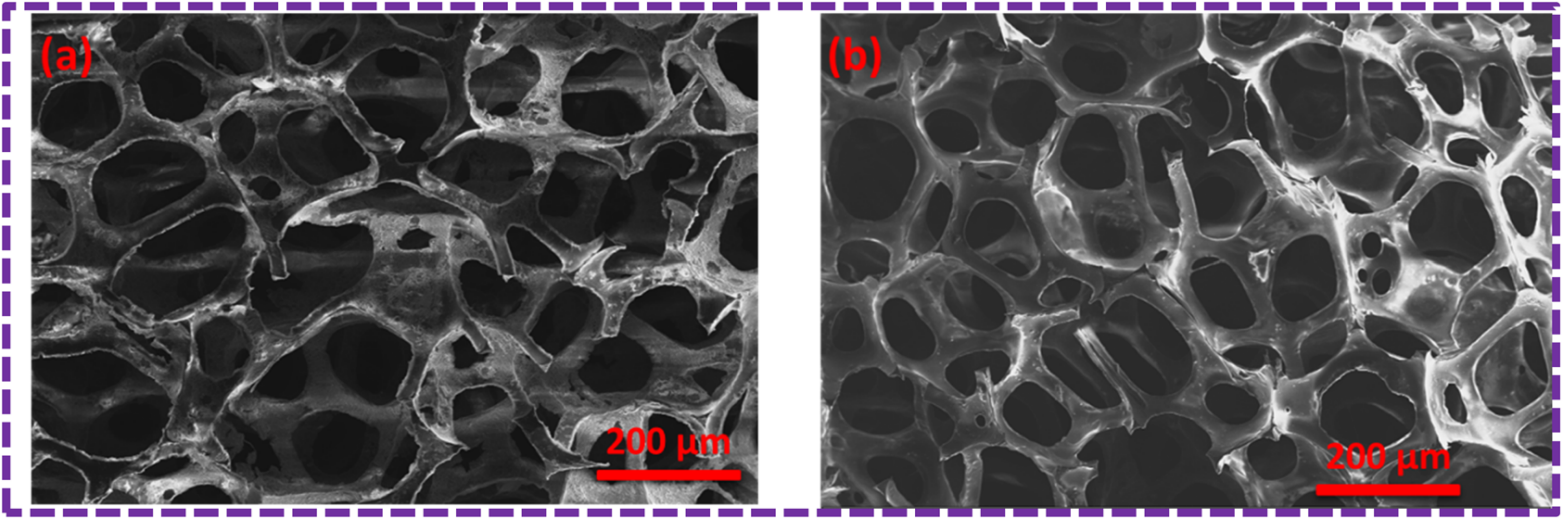
(a) SEM image of the ZnS/MM/PVA-modified PU foam before the oil sorption test. (b) SEM image of the ZnS/MM/PVA-modified PU foam after the oil sorption test.

The surface modification of the PU foam with the ZnS/MM/PVA nanocomposite was observed with FESEM/EDX before the sorption test. These foams possess a void space and surface area to absorb the used oil. Fig. 8a and b compares the morphology of the nanocomposite before and after the oil sorption test. We observed that after surface modification, the surface becomes rough due to the coating of ZnS/MM/PVA. Due to amplification or enlargement of the solid–liquid interactions, this introduced irregularity to the foam influences its interaction with a wetting or nonwetting liquid. The uniform cell structure and spherical shape of the modified foam sorbent indicate that the incorporation of ZnS/MM/PVA into the foam did not result in the destruction of the PU foam structure.^36^Fig. 8a shows that the modified PU foam has ZnS, MM and PVA. Fig. 8b shows that after 10 cycles of the oil sorption test, the surface stability of the PU foam is quite evident.

**Fig. 9:**
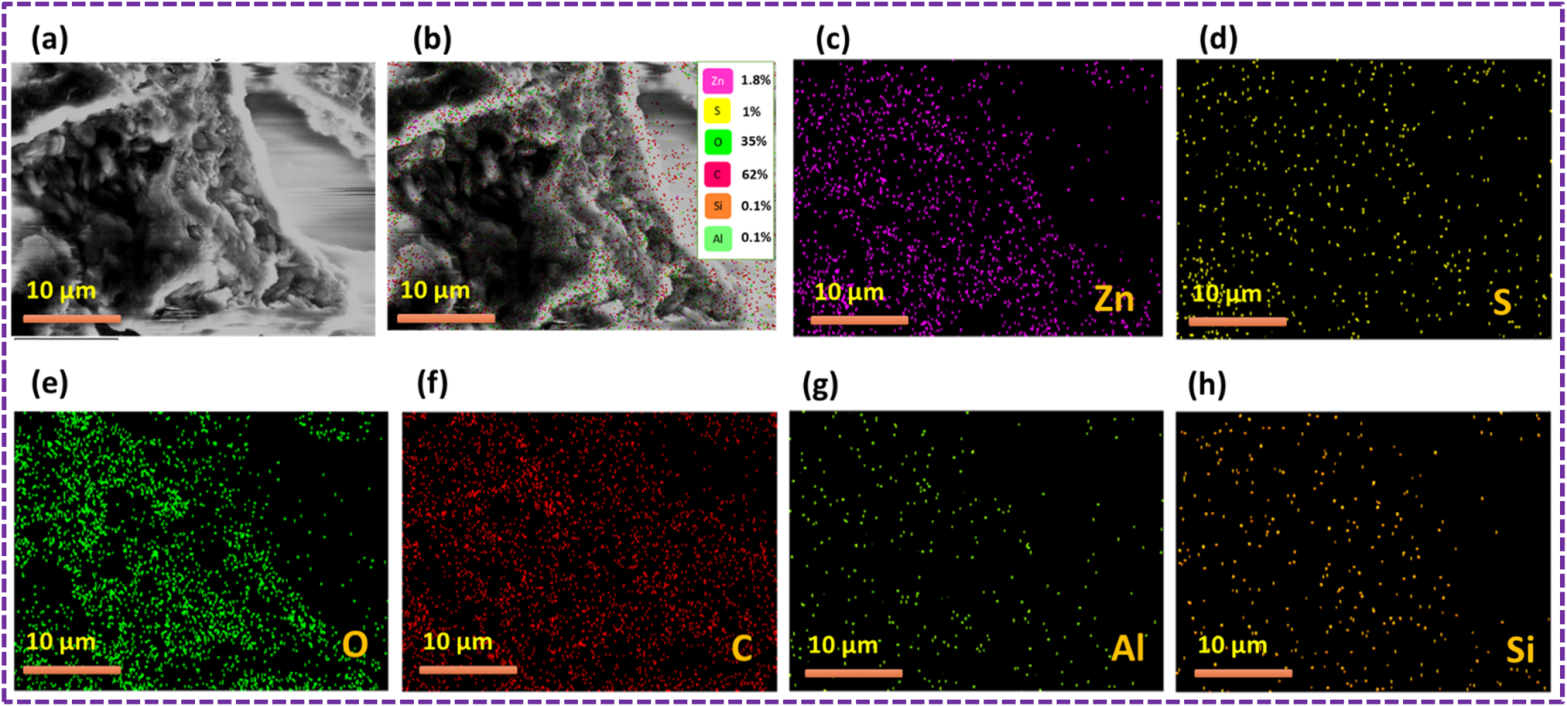
SEM image of the ZnS/MM/PVA-modified PU foam; (a and b) EDX mapping and elemental analysis, including elemental analysis of (c) Zn, (d) S, (e) O, (f) C, (g) Al, and (h) Si before the oil sorption test.

Fig. 9 shows the results of the elemental mapping before the oil sorption test, which indicate the presence of Zn, S, C, O, Si and Al, suggesting that the ZnS/MM/PVA nanoparticles are present. Throughout the nanocomposite, Zn, S, C, O, Si and Al atoms were found to be evenly dispersed.

**Fig. 10:**
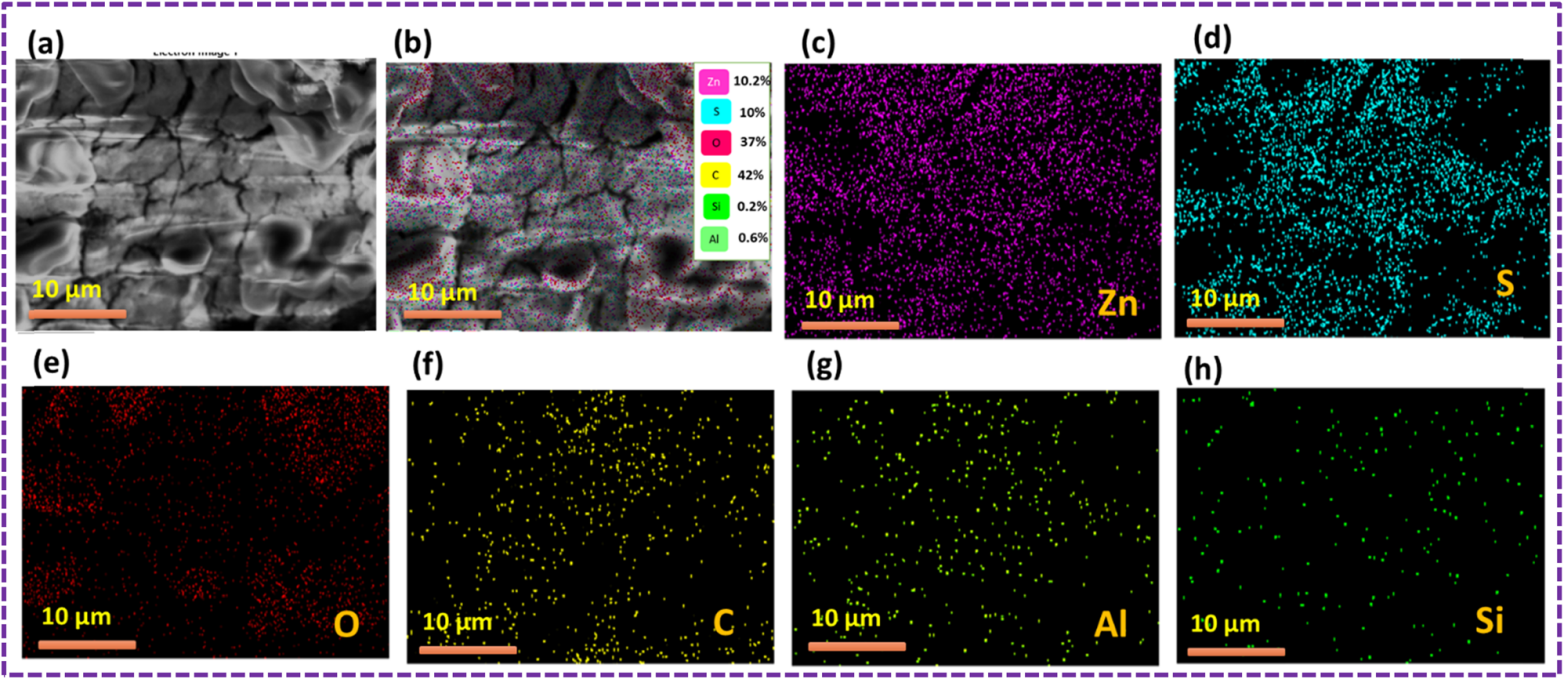
SEM image of the ZnS/MM/PVA-modified PU foam; (a and b) EDX mapping and elemental analysis, including elemental analysis of (c) Zn, (d) S, (e) O, (f) C, (g) Al, and (h) Si after the oil sorption test.

The results of the oil sorption test, as depicted in Fig. 10, revealed the presence of Zn, S, C, O, Si, and Al using elemental mapping. This suggests the existence of the ZnS/MM/PVA nanoparticles. The nanocomposite exhibited a uniform dispersion of Zn, S, C, O, Si, and Al atoms throughout the material. The carbon content was increased after conducting an oil sorption test.

### FT-IR

**Fig. 11:**
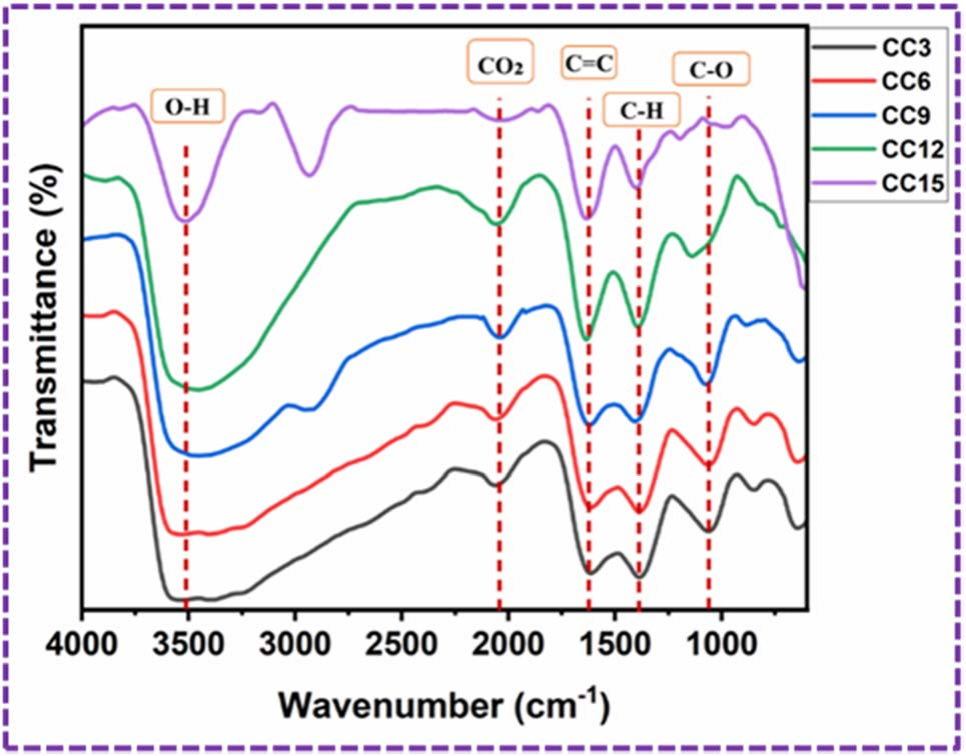
FT-IR spectra of the CC3, CC6, CC9, CC12 and CC15-modified PU foams.

The FT-IR spectra of the CC3, CC6, CC9, CC12, and CC15-modified PU foams are shown in Fig. 11. The observed wide absorption peaks at 3510.9 cm^−1^ for the modified PU foam are caused by the O–H bond stretching vibrations of PVA. The broadness of the band peak decreases when the quantity of CC increased.^37^ The peaks found at 2041 cm^−1^ are due to the presence of CO_2_ molecules from the atmosphere.^38^ The presence of the functional group as the double bond C=C indicates the existence of the aromatic stretching vibration at 1627.9 cm^−1^.^39^ The peak seen at 1390.7 cm^−1^ corresponds to the carboxyl (C=O) and methylene groups.^38^ The peak observed at 1058.1 cm^−1^ is caused by the existence of the C–O functional group, specifically the stretching of C–O bonds in the acetyl groups. The peak is shifted towards the left when the quantity of CC increased.^40^

**Fig. 12:**
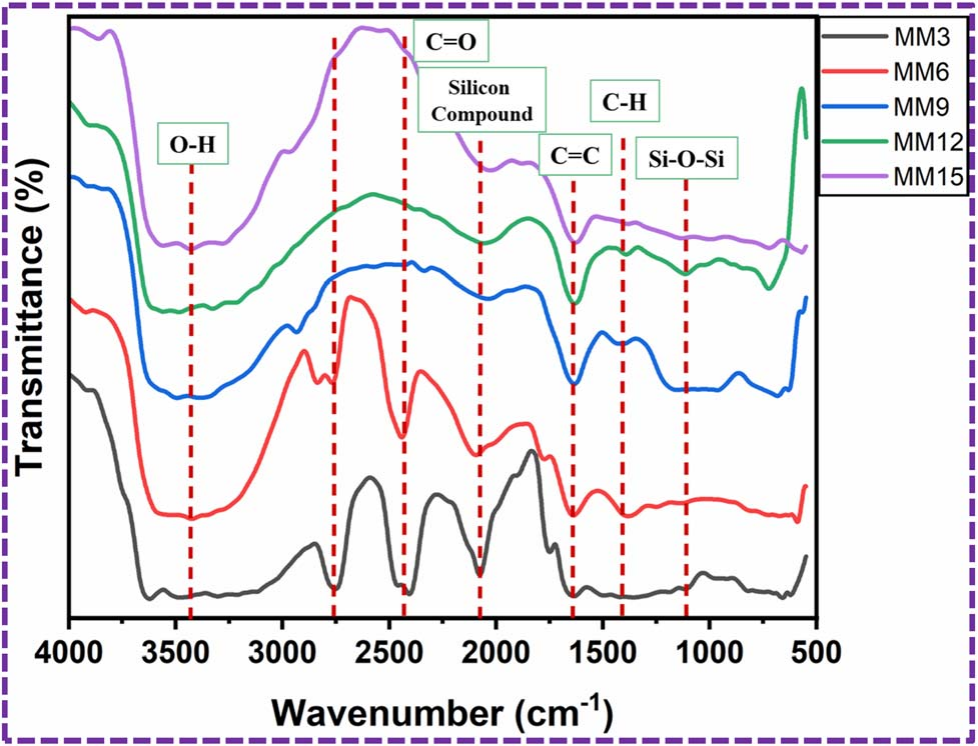
FT-IR spectra of the MM3, MM6, MM9, MM12 and MM15-modified PU foams.

The FT-IR spectra of the MM3, MM6, MM9, MM12, and MM15-modified PU foams are shown in Fig. 12. The observed wide absorption peaks at 3428.6 cm^−1^ for the modified PU foam are caused by O–H bonds of the stretching vibrations of water, and the broadness of the band peak decrease when the quantity of MM increased.^37^ The band at 2758.3 cm^−1^ indicated the carboxyl acid.^41^ In addition, a peak at around 2422.4 cm^−1^ was assigned to the C=O stretching vibrations.^42^ When the quantity of MM increased, the peaks at 2758.3 cm^−1^, 2422.2 cm^−1^ and 2076 cm^−1^ disappeared. The presence of the C=C double bond is indicated by the existence of the aromatic stretching vibration at 1633.9 cm^−1^.^39^ The peak seen at 1405.7 cm^−1^ corresponds to the C–H bending vibration of CH_2_.^40,43^ The band peak at 1110 cm^−1^ indicates the presence of the bonding vibration of Si–O–Si.^33,39,44,45^

The peaks of the pristine PU foam correspond to the N–H stretching vibration, C–H stretching vibration of the CH_2_ group, C=O bond, C–N stretching band, C=O stretching band, and C–O stretching vibration.^46^

### Viscosity of used oil

The viscosities of the commercial and used oils at 25 °C and 40 °C temperature are shown in Table 1.

**Table 1:** Viscosity of the used oil measured at room temperature

Sr. No.	Oils	Temperature (°C)	Viscosity of commercial oil (Pa)	Viscosity of used oil (Pa)
1	Vegetable oil	25	0.0462 (ref. 47)	0.0957
		40	0.0245 (ref. 47)	0.0527
2	Engine/luba oil	25	0.1422 (ref. 48)	0.1551
		40	0.0559 (ref. 48)	0.07706
3	Pump/hydraulic oil	25	0.0242 (ref. 49)	0.2777
		40	0.0506 (ref. 49)	0.119
4	Gear oil	25	0.2365 (ref. 50)	0.2796
		40	0.1454 (ref. 50)	0.1652

### TGA

**Fig. 13:**
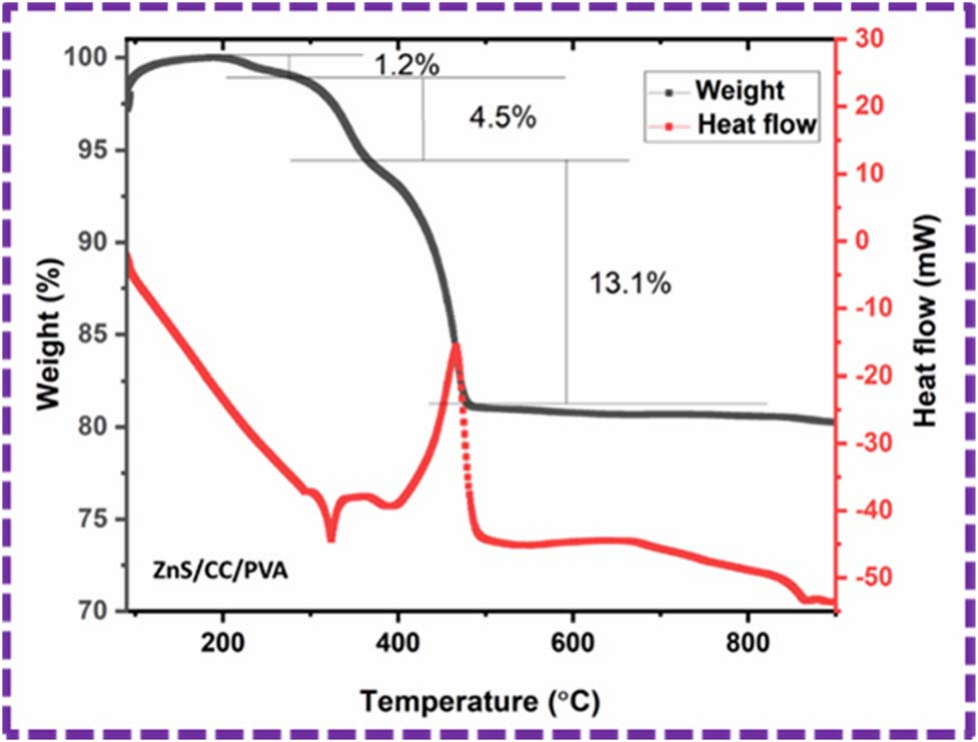
TGA (weight%) and DSC (heat flow) graph of the ZnS/CC/PVA composite in the temperature range of 49–900 °C.

Thermal stability of the ZnS/CC/PVA composite was evaluated using TGA, as shown in Fig. 13. The composite material demonstrated a three-stage decomposition process, leading to three distinct phases of weight reduction when subjected to a nitrogen environment. The first degradation stage was detected in the temperature range between 214–295 °C, and the corresponding weight losses for the composite samples were observed to be 1.2%. The observed decline can be attributed to the process of moisture evaporation, and is related to the water loss.^51^ The second degradation happened within the temperature range of 295–370 °C, having weight losses of 4.5%. This degradation is attributed to the decomposition temperature of the PVA structure.^52,53^ The maximum degradation happened at the third phase within the temperature range of 370–481 °C, resulting in a weight loss of 13.1% that represents the ash and fixed carbon content.^51^ The final weight of the sample is 80.14%.

The differential scanning calorimetry (DSC) analysis associated with the TGA of ZnS/CC/PVA exhibited the heat flow change with temperature, and was used to determine the melting and crystallization temperatures. The melting^54^ and crystallization^55^ temperatures of the composite were evaluated as 322 °C and 465 °C, respectively.

**Fig. 14:**
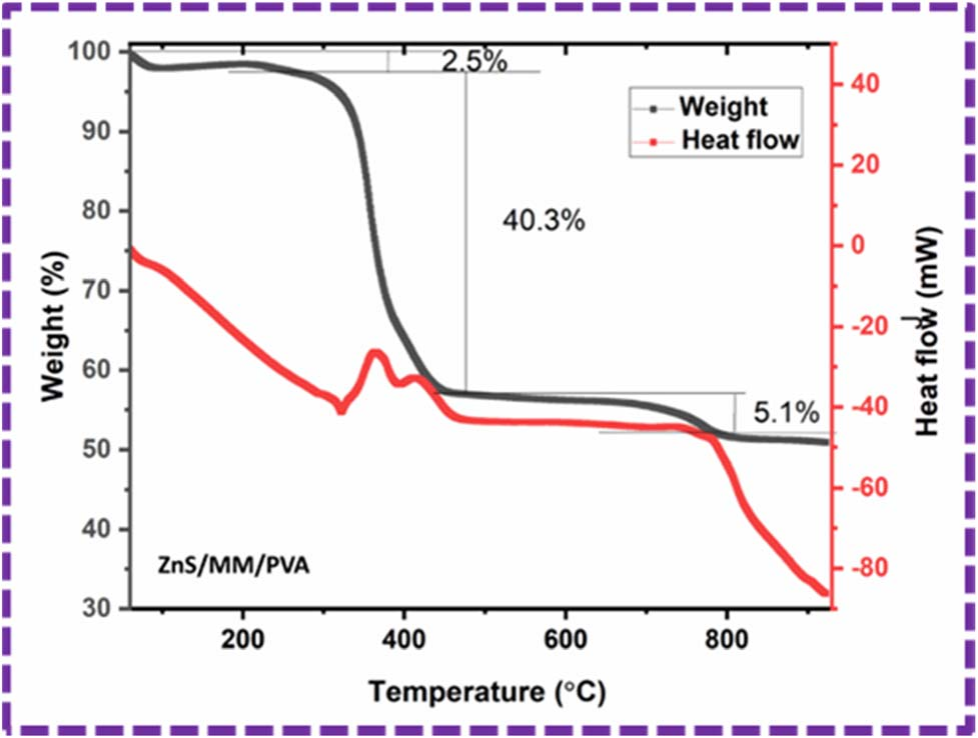
TGA (weight%) and DSC (heat flow) graph of the ZnS/MM/PVA composite in the temperature range of 49–900 °C.

Thermal stability of the ZnS/MM/PVA composite was evaluated using TGA, as shown in Fig. 14. The composite material demonstrated a three-stage decomposition process, leading to three distinct phases of weight reduction when subjected to a nitrogen environment. The first degradation stage was detected within the temperature range of 59–279 °C, and the corresponding weight losses for the composite samples were observed to be 2.5%. The observed decline can be attributed to the process of moisture evaporation and related to the water loss.^51^ The maximum degradation happened at the second phase within the temperature range of 279–551 °C, having weight losses of 40.3%. The second degradation is caused by the elimination of water molecules that were strongly attached to the octahedral cations, as well as the removal of silanol groups from MM^56^ and the thermal decomposition of the precursor into ZnS particles.^57^ This degradation is attributed to the decomposition temperature of the PVA structure.^52,53^ The third phase degradation occurs within the temperature range of 551–804 °C, resulting in a weight loss of 5.1%. This weight loss can be ascribed to the emission of CO_2_ as a result of decomposition.^58^ The final weight of the sample is 50.91%.

The differential scanning calorimetry (DSC) analysis associated with the TGA of ZnS/MM/PVA exhibited the heat flow change with temperature, and was used to determine the melting and crystallization temperatures. The melting^54^ and crystallization^55^ temperatures of the composite were evaluated as 321 °C and 411 °C, respectively.

### XPS

**Fig. 15:**
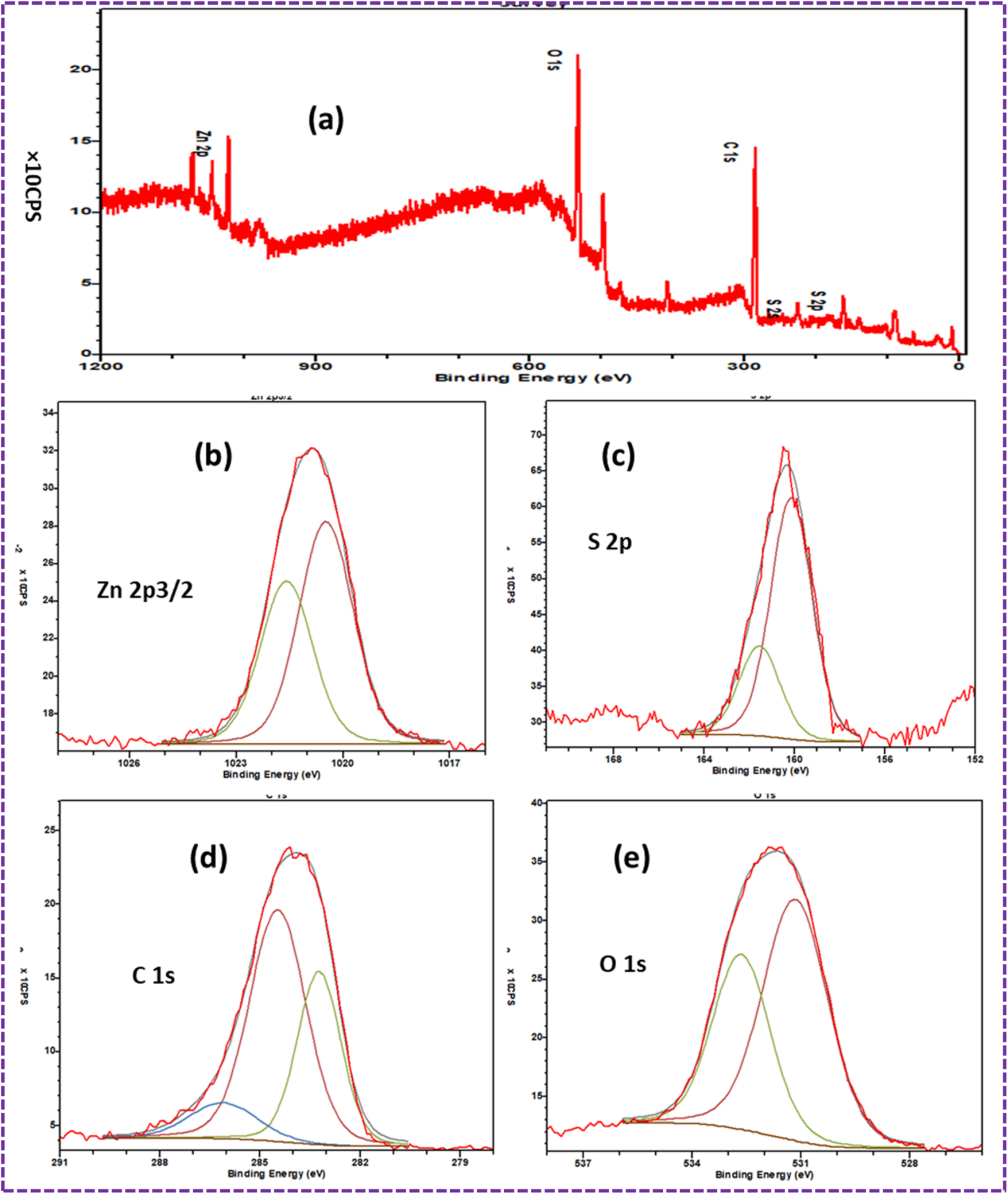
XPS spectra of the dried ZnS/CC/PVA-modified PU foam: (a) survey spectrum indicating the presence of Zn, S, O, C, and (b) Zn 2p_3/2_, (c) S 2p, (d) O 1s, and (e) C 1s, respectively.

The survey spectra of the ZnS/CC/PVA-modified PU foam composites are displayed in Fig. 15a. Particularly, the peaks corresponding to Zn 2p_3/2_, O 1s, C 1s, and S 2p are readily discernible. The binding energy of Zn 2p_3/2_ observed at 1020.5 eV indicates the presence of Zn^2+^ in ZnS.^59^ The 1022.0 eV binding energy of Zn 2p^3/2^ can be attributed to the Zn–S bond^60^ (Fig. 15b). The S 2p peaks at 160 eV, 160.4 eV and 161.7 eV can be attributed to the S bond in ZnS (S–) shown in Fig. 15c.^61^ The composites' high-resolution C 1s XPS spectrum is illustrated in Fig. 15d. The peaks indicated the existence of C–C bonds at about 283.2 eV and C–O–C bonds at around 284.9 eV, which both correlate to the accumulation of the carbon content on the surface of the coating. A low-intensity wide peak, indicative of carboxylate bonds, was found at around 286.3 eV.^62^ The peak at 530 eV indicated the presence of substances that collected on the surface of the coating.^62^ The O 1s peak had the highest intensity at the binding energy of 531.6 eV, corresponding to C=O,^63^ in comparison to the other elements shown in Fig. 15e. The broad peak is composed of the OH group of PVA. Due to the large number of –OH groups in PVA, the O 1s intensity was the highest.^64^ The peak at 532.8 eV corresponds to the C–O–C/C–OH bond.^63^

### Density

The densities of the PU foam are directly related with its absorption capacity,^33^ and calculated by eqn (3). The initial density of the pristine PU foam is 0.05 g cm^−3^. However, the density of the foam rises to 1.94 g cm^−3^ and 0.16 g cm^−3^ when increasing concentrations of CC and MM were added. This indicates the good adherence of ZnS/CC/PVA and ZnS/MM/PVA to the surface of the PU foam by the process of dip coating.

### Sorption test

Used oils (such as vegetable oil, hydraulic oil, gear oil and lube oil) are used for the oil sorption test. Firstly, we measured the ZnS, ZnS/PVA-modified PU foams for comparison. The sorption capacities of the pristine PU foam in vegetable oil, hydraulic oil, gear oil and lube oil are 3.2, 2.5, 2.1 and 1.9 g g^−1^, respectively. The sorption capacities of ZnS in vegetable oil, hydraulic oil, gear oil and lube oil are 7.82, 6.91, 5.39, and 6.65 g g^−1^, respectively. The sorption capacities of ZnS/PVA in vegetable oil, hydraulic oil, gear oil and lube oil are 9.25, 7.23, 6.10, and 8.24 g g^−1^, respectively. The sorption capacities of CC in vegetable oil, hydraulic oil, gear oil and lube oil are 7.7, 6.19, 7.75 and 6.5 g g^−1^, respectively. The sorption capacities of CC/PVA in vegetable oil, hydraulic oil, gear oil and lube oil are 10.2, 10.5, 9, and 9.3 g g^−1^, respectively. The sorption capacities of ZnS/CC in vegetable oil, hydraulic oil, gear oil and lube oil are 7.8, 6.61, 8, and 8.6 g g^−1^, respectively. These sorption capacities of pristine PU foam, ZnS, ZnS/PVA, CC, CC/PVA and ZnS/CC-modified PU foam are compared in Table 2 and Table 3.

**Table 2:** Oil sorption capacity (g g^−1^) of the ZnS/CC/PVA-modified PU foam

Sr. No.	Oils	Vegetable (g g^−1^)	Pump/hydraulic (g g^−1^)	Engine/lube (g g^−1^)	Gear (g g^−1^)
	Material				
1	CC3	12.16 ± 1	13.23 ± 1	13.11 ± 1	12.89 ± 1
2	CC6	14.01 ± 1	13.96 ± 1	13.43 ± 1	16.13 ± 1
3	CC9	15.2 ± 1	12.67 ± 1	14.33 ± 1	16.78 ± 1
4	CC12	14.62 ± 1	12.32 ± 1	12.92 ± 1	15.78 ± 1
5	CC15	11.87 ± 1	10.97 ± 1	12.21 ± 1	11.43 ± 1

**Table 3:** Oil sorption capacity (g g^−1^) of the ZnS/MM/PVA-modified PU foam

Sr. No.	Oil	Vegetable (g g^−1^)	Pump/hydraulic (g g^−1^)	Engine/lube (g g^−1^)	Gear (g g^−1^)
	Materials				
1	MM3	10.48 ± 1	10.47 ± 1	11.95 ± 1	10.84 ± 1
2	MM6	14.49 ± 1	11.28 ± 1	12.28 ± 1	12.21 ± 1
3	MM9	15 ± 1	13.31 ± 1	13.13 ± 1	13.52 ± 1
4	MM12	15.9 ± 1	13.5 ± 1	13.5 ± 1	16 ± 1
5	MM15	14.5 ± 1	14.5 ± 1	13.4 ± 1	14.8 ± 1

**Fig. 16:**
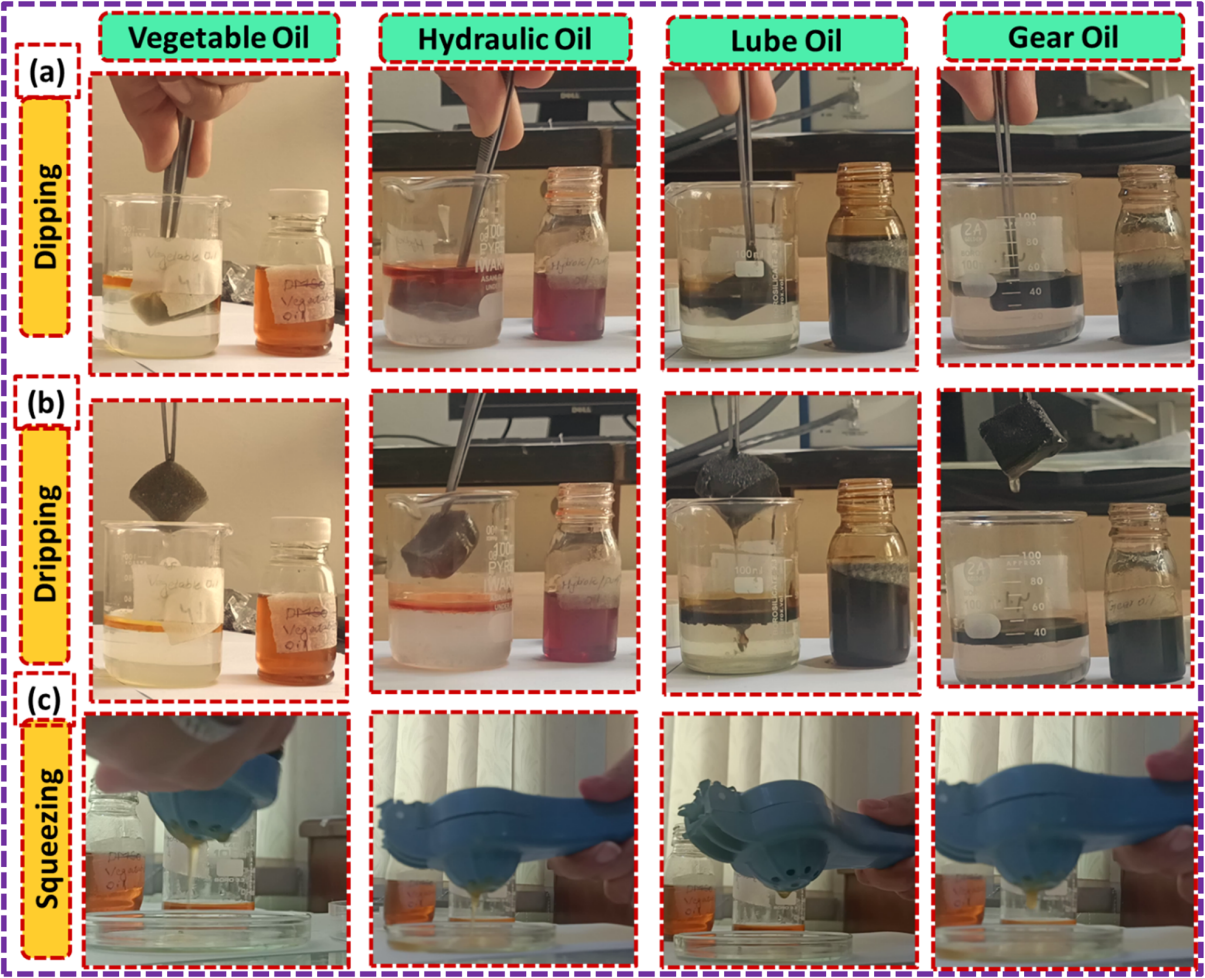
Oil sorption capacity test (g g^−1^) of the modified PU foam: (a) dipping, (b) dripping, and (c) squeezing.

**Fig. 17:**
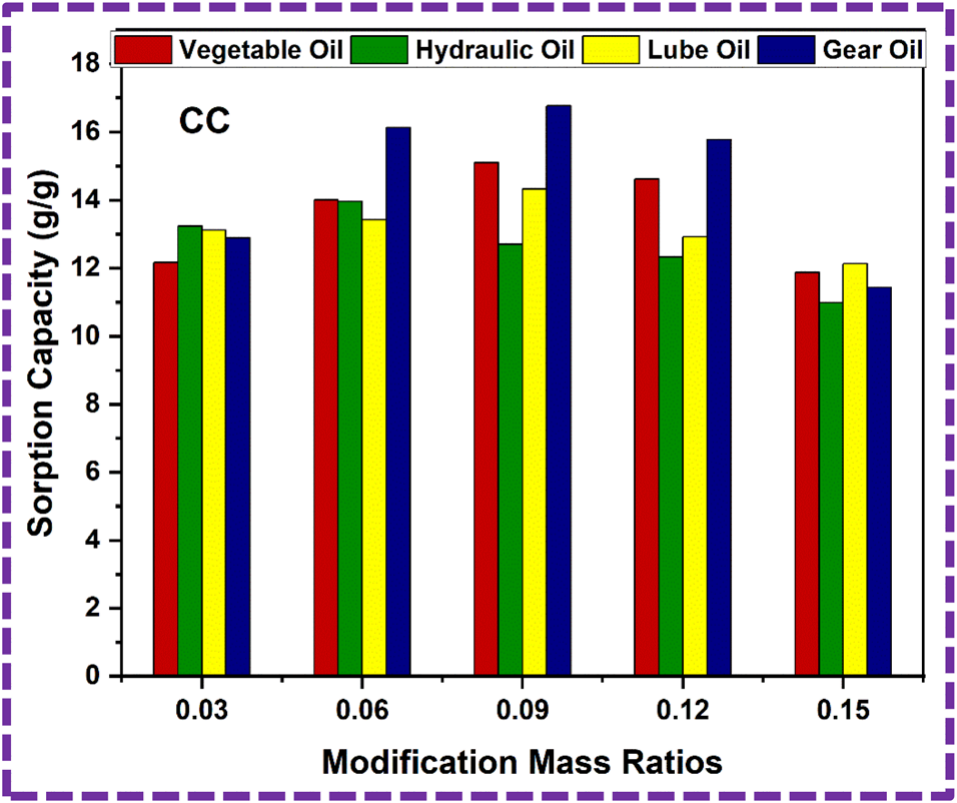
Sorption capacity graph of the ZnS/CC/PVA-modified PU foam.

Now, we dip the ZnS/CC/PVA-modified PU foam into the oil water solution, and then drip it for approximately two minutes, as shown in Fig. 16. The modified PU foam has greatly improved oil sorption capacity, as shown in ESI Videos (V1–V4).† The ZnS/CC/PVA-modified PU foam has excellent sorption capability, and can easily adsorb oil droplets above or below the water surface (when the foam was dipped under water). When oil is adsorbed in the modified PU foam pores, the pore volume of the PU foams was completely filled and also adsorbed on the outer surface of the foam. Its absorption capacities vary with the usage of different oils. The sorption capacity of the modified PU foams is shown in Table 2. CC9 has the high sorption capacity compared to the other modified foams, which is calculated by eqn (4). The sorption capacity graph of the modified PU foam is shown in Fig. 17.

**Fig. 18:**
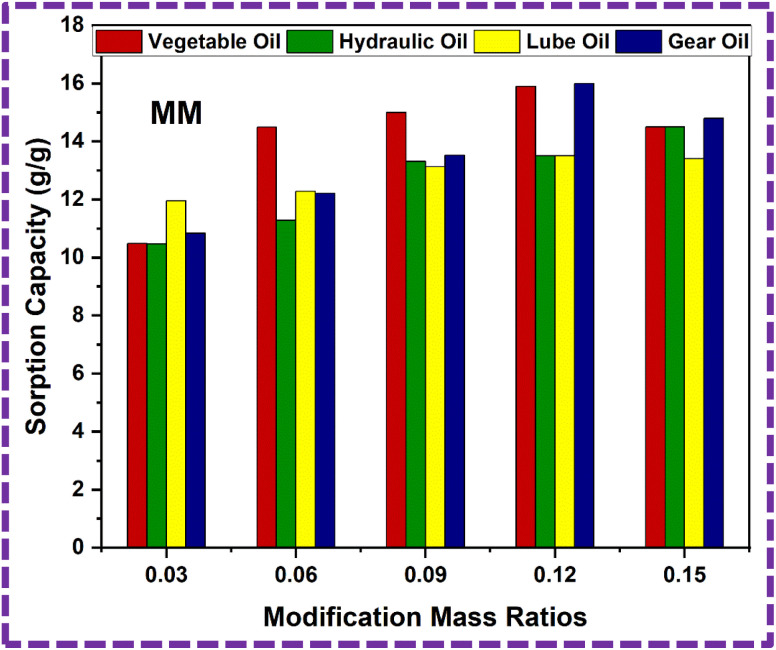
Sorption capacity graph of the ZnS/MM/PVA-modified PU foam.

The modified PU foam has greatly improved oil sorption capacity, as shown in the ESI Video.† The ZnS/MM/PVA-modified PU foam has excellent sorption capability, and can easily absorb oil droplets above or below the water surface (when the foam was dipped under water by tweezers). When oil is adsorbed in the modified PU foam pores, the pore volume of the PU foams was completely filled and also adsorbed on the outer surface of the foam. Its absorption capacities vary with the usage of different oils. The sorption capacity of the modified PU foams is shown in Table 3. MM12 has the high sorption capacity compared to the other modified foams, which is calculated by eqn (3). The sorption capacity graph of the modified PU foam is shown in Fig. 18. The MM is more adhesive than CC, and the surface area of CC is greater than that of MM.^65^ This is why the sorption capacity of MM is less than that of CC.

### Reusability

**Fig. 19:**
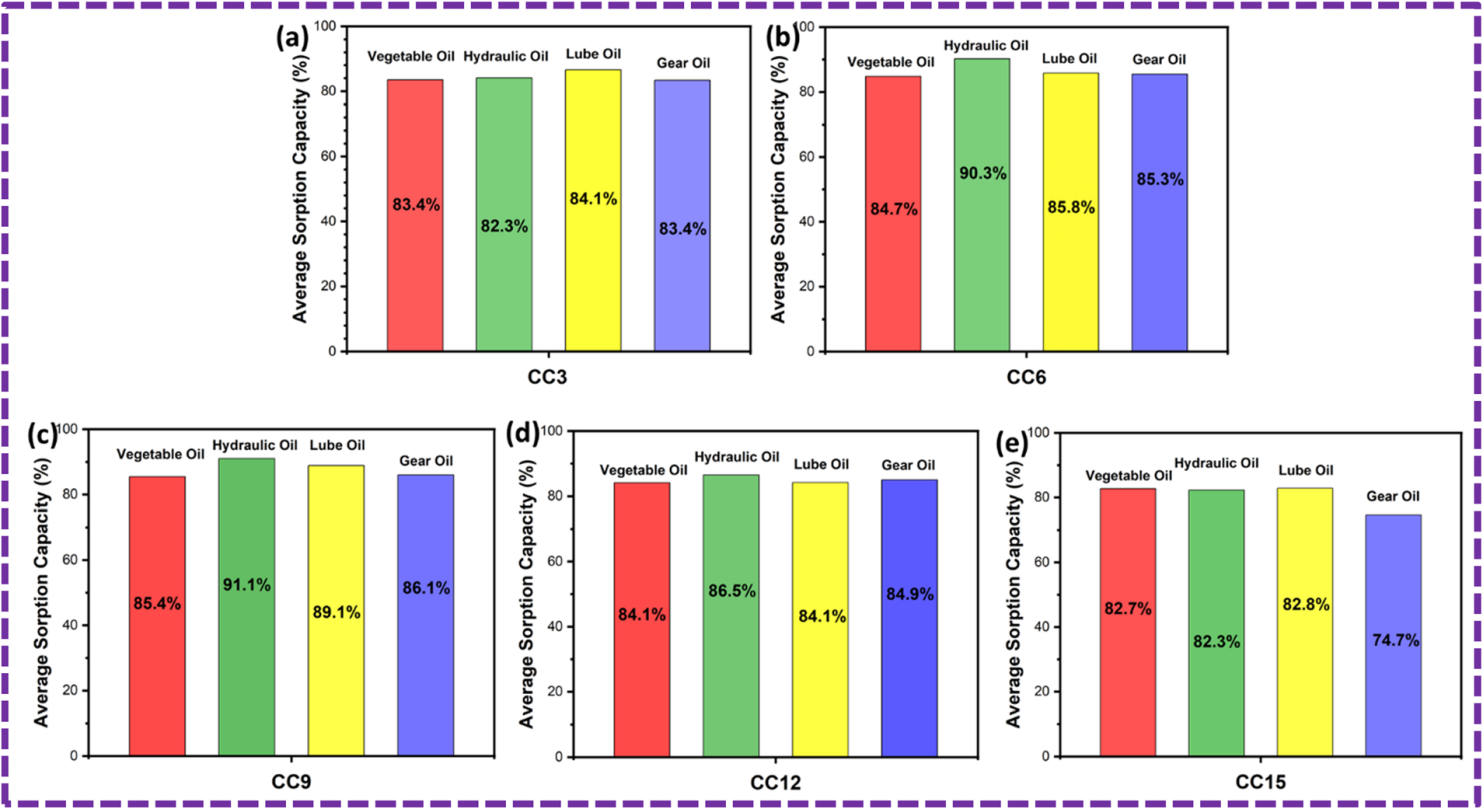
Average sorption capacity after 10 cycles of the ZnS/CC/PVA-modified PU foam.

**Fig. 20:**
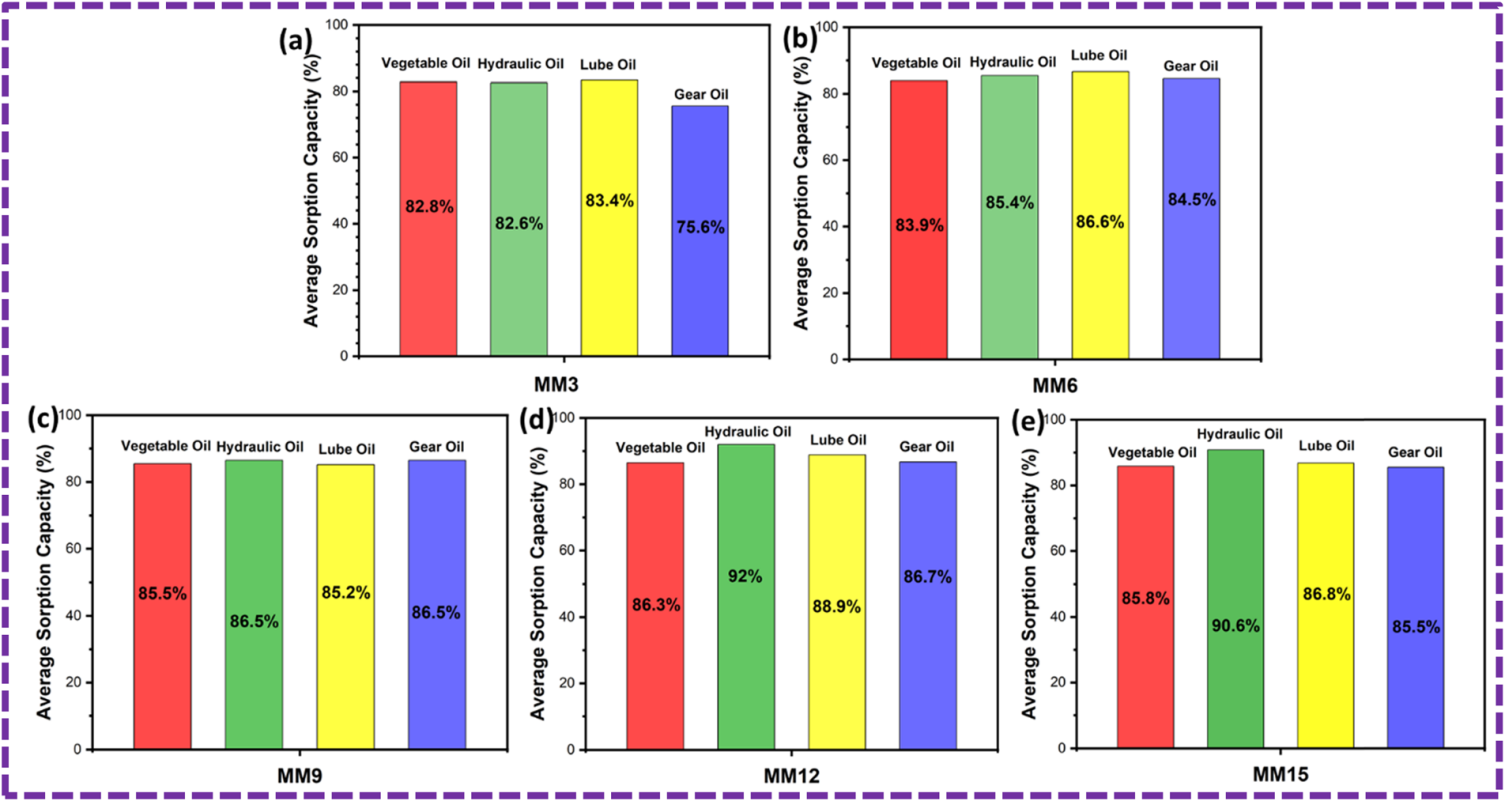
Average sorption capacity after 10 cycles of the ZnS/MM/PVA-modified PU foam.

The ZnS/CC/PVA and ZnS/MM/PVA-modified PU foams have good elasticity, and the oil can be easily removed by softly squeezing the foam (Fig. 19 and 20). However, the PU foam does not completely remove all of the oil when it is squeezed. After the absorption or desorption cycle, a small portion of the nanocomposite was detached from the modified PU foam, but this does not decrease the oil absorption ability. The recovery rate of the vegetable, hydraulic, lube, and gear oils was 73%, 68%, 70% and 67%, respectively, by the ZnS/CC/PVA-modified PU foam. The recovery rate of the vegetable, hydraulic, lube, and gear oils was 71%, 66%, 79% and 63%, respectively, by the ZnS/MM/PVA-modified PU foam. Table 4 shows the comparative study with the previous literature.

**Table 4:** Literature for the oil sorption capacities of the modified PU foams

Sr. No.	Materials	Synthesis method	Oils	Volume of PU foam (cm^3^)	Sorption capacity (g g^−1^)	Year	Ref.
1	SiO_2_-PU	Immersion process	Motor oil	13.5	103 ± 3	2014	19
			Diesel oil		95 ± 3		
2	PU-TiO_2_ (TPU-GO-TDA)	Ultrasonication	Engine oil		13.6 ± 1	2018	36
			Crude oil		13.2 ± 1		
			Silicon oil		14.2 ± 1		
			Engine oil		25.83		
3	LPU with F-SiO_2_	Immersion process	Soybean oil	0.25	2.1	2023	66
			Pump oil		2.1		
			Crude oil		2		
4	ZnO-PC	Immersion process	Diesel oil	1	14.78	2023	67
5	ZnS/CC/PVA	Coprecipitation method	Vegetable oil	4	15.2 ± 1		This work
			Pump/hydraulic oil		12.67 ± 1		
			Engine/lube oil		14.33 ± 1		
			Gear oil		16.78 ± 1		
6	ZnS/MM/PVA	Coprecipitation method	Vegetable oil	4	15.9 ± 1		This work
			Pump/hydraulic oil		13.5 ± 1		
			Engine/lube oil		13.5 ± 1		
			Gear oil		16 ± 1		

### Mechanical stability

**Fig. 21:**
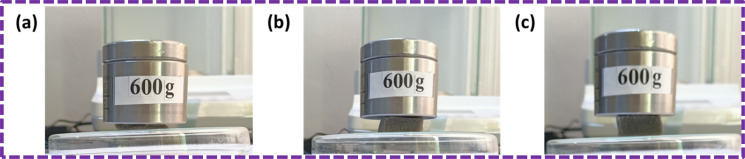
Mechanical stability of the (a) pristine PU foam, (b) ZnS/CC/PVA and (c) ZnS/MM/PVA-modified PU foams.

The weight capacity of the PU foam increased as a result of the coating with ZnS/CC/PVA and ZnS/MM/PVA nanocomposites. The ZnS/CC/PVA and ZnS/MM/PVA-modified PU foams have exceptional mechanical durability. Thus, when putting a 600 g weight onto a pristine PU foam with dimensions of 2 cm × 2 cm × 1 cm, the sponge exhibited significant compression and deformation. In contrast, the ZnS/CC/PVA and ZnS/MM/PVA modified PU foams exhibited remarkable mechanical strength, enabling it to retain its original shape even under the same weight load (shown in Fig. 21). The mechanical strength of the ZnS/CC/PVA and ZnS/MM/PVA-modified PU foams exhibits different characteristics.^68^

## Conclusions

In this study, ZnS/CC/PVA and ZnS/MM/PVA were used to improve the oil sorption capacity of the PU foam. The ZnS/CC/PVA and ZnS/MM/PVA-modified PU foams with different concentrations of CC and MM were modified by coprecipitation method for oil spill cleanup. XRD, FESEM, FTIR, TGA and XPS confirm the successful preparation of the ZnS/CC/PVA and ZnS/MM/PVA-modified PU foams. Used oils (such as vegetable, lube, hydraulic and gear oils with different viscosities) were selected for the sorption capacity test. The ZnS/CC/PVA and ZnS/MM/PVA-modified PU foams have exceptional mechanical strength and high sorption capacity, absorbing gear oil that is 16.78 and 16 times its own weight, respectively. After that, it can be reused 10 times by squeezing. Its sorption capacity remains essentially the same. Furthermore, it possesses excellent cyclic stability and reusability. The experimental results demonstrated that the ZnS/CC/PVA and ZnS/MM/PVA-modified PU foam sorbents hold great promise for oil spill cleanup and recovery in oil-water systems.

## Data availability

The data supporting this article have been included as part of the ESI.†

## Author contributions

The manuscript was written through contributions of all authors. All authors have given approval to the final version of the manuscript.

## Conflicts of interest

There are no conflicts to declare.

## Supplementary Material

RA-014-D4RA03924F-s001

RA-014-D4RA03924F-s002

RA-014-D4RA03924F-s003

RA-014-D4RA03924F-s004
